# Effects of *Levilactobacillus brevis* GKEX supplementation on exercise performance and fatigue resistance in mice

**DOI:** 10.3389/fnut.2025.1625645

**Published:** 2025-10-14

**Authors:** Tzu-Chun Lin, Chin-Chu Chen, You-Shan Tsai, Shih-Wei Lin, Chi-Chang Huang, Yi-Ju Hsu

**Affiliations:** ^1^Biotech Research Institute, Grape King Bio Ltd., Taoyuan, Taiwan; ^2^Department of Food Science, Nutrition, and Nutraceutical Biotechnology, Shih Chien University, Taipei, Taiwan; ^3^Institute of Food Science and Technology, National Taiwan University, Taipei, Taiwan; ^4^Graduate Institute of Sports Science, National Taiwan Sport University, Taoyuan, Taiwan; ^5^Tajen University, Pingtung, Taiwan

**Keywords:** *Levilactobacillus brevis* GKEX, exercise performance, fatigue resistance, lactate, blood urea nitrogen, short-chain fatty-acid-producing bacteria

## Abstract

**Background:**

Exercise-induced muscle fatigue is a major challenge for athletes. Our previous study indicated that *Levilactobacillus brevis* GKEX improved endurance and reduced fatigue in mice. This study aimed to further evaluate the effects of different preparations of *L. brevis* GKEX on exercise performance and fatigue resistance.

**Methods:**

Eight-week-old male ICR mice were divided into six groups (*n* = 8): vehicle, BCAA, low-dose GKEX (0.0615 mg/day), high-dose GKEX (0.615 mg/day), heat-killed GKEX (0.615 mg/day), and freeze-killed GKEX (0.615 mg/day). Treatments lasted for four consecutive weeks. Exercise performance, fatigue-related biochemical markers, and gut microbiota composition were assessed.

**Results:**

Compared with BCAA, *L. brevis* GKEX significantly improved aerobic performance, including forelimb grip strength and running-exhaustion time. It enhanced lactate clearance and glycogen storage in the liver and muscles while reducing lactate production and blood urea nitrogen levels after exercise. *L. brevis* GKEX supplementation also increased key short-chain fatty-acid–producing bacteria in the intestines.

**Conclusion:**

Oral administration of different doses of live and dead *L. brevis* GKEX promoted exercise performance and ameliorated fatigue, especially live GKEX. These findings suggest that *L. brevis* GKEX may serve as an ergogenic aid for athletes and support broader applications across various product forms.

## 1 Introduction

When the body experiences energy depletion or tissue damage, it may result in a sensation of fatigue primarily caused by exercise, inadequate sleep, illness, or other factors. Exercise-induced muscle fatigue is one of the most prevalent and easily identifiable types of fatigue. During exercise, the body utilizes various energy sources, including liver glycogen and glucose. When energy expenditure, coupled with the accumulation of lactate, can lead to feelings of weakness or muscle pain, it ultimately affects exercise performance (1). Prolonged muscle fatigue may even lead to neuromuscular disorders, metabolic diseases, and other adverse health conditions (2). Athletes and other individuals typically use various supplements, such as vitamins, traditional herbs, and minerals, to alleviate fatigue and improve their athletic performance. However, consuming supplements without scientific validation of their efficacy can potentially increase the burden on the body, leading to side effects, such as allergic reactions or digestive problems.

Probiotics are defined as non-pathogenic microorganisms that, when ingested, exert a positive influence on the host’s health or physiology (3). Among the most common probiotics are lactic acid bacteria, with *Lactobacillus* being the most prevalent genus. Studies indicate that *Lactobacilli* have proven functions, including reducing allergic reactions (4) and exerting antioxidant and anti-aging effects (5). They also have applications in intestinal inflammatory and infectious diseases (6), in addition to being used as an adjuvant for cancer prevention (7). Thus, it is evident that *Lactobacilli* have a beneficial impact on the overall health maintenance and promotion of bodily functions.

In recent years, there has been a growing interest in *Lactobacillus* supplements for reducing post-exercise fatigue while enhancing exercise performance. For instance, supplementation with *Limosilactobacillus reuteri* (formerly *Lactobacillus reuteri*) ID-D01 enhanced exercise endurance and muscle growth in C57BL/6 mice (8). Additionally, supplementation with heat-killed *Lactiplantibacillus plantarum* (formerly *Lactobacillus plantarum*) TWK10 has been found to significantly reduce physical fatigue, improve exercise endurance capacity, and increase overall muscle weight in healthy humans (9). Specifically, supplementation with *Lactobacillus salivarius* SA-03 increases muscle strength, decreases fatigue levels, and also affects the distribution of intestinal microbiota (10).

*Levilactobacillus brevis* (*L. brevis*) GKEX is a heterofermentative, Gram-positive lactic acid bacterium isolated from douchi (fermented black soybeans). The strain was identified and selected by Grape King Bio Ltd. (Taoyuan, Taiwan) based on its potential probiotic properties, including acid and bile salt tolerance. Our previous study demonstrated that supplementation with *L. brevis* GKEX significantly enhanced exercise endurance in a murine model, reduced fatigue-related biomarkers such as lactate and blood urea nitrogen levels, and increased glycogen content in the liver and muscles (11). Additionally, in our recent human trials, a six-week resistance exercise training program combined with *L. brevis* GKEX supplementation resulted in increased muscle mass and improved strength performance (12). These findings highlight the strain’s potential as a novel probiotic in exercise supplementation. Furthermore, non-viable probiotics offer several practical advantages, such as greater safety, stability, and shelf life, especially in immunocompromised populations (13). Therefore, this study aims to further investigate the fatigue-relieving and performance-enhancing effects of both live and non-viable forms of *L. brevis* GKEX, as well as its dose-dependent efficacy for broader applications. This will be evaluated through endurance testing, the regulation of fatigue-related biomarkers, and gut microbiota analysis.

## 2 Materials and methods

### 2.1 Sample preparation

The experimental strain of *L. brevis* GKEX provided by Grape King Bio Ltd. (Taoyuan, Taiwan) was originally isolated from douchi (fermented black soybeans) and preserved at the National Institute of Technology and Evaluation, Biological Resource Center (NBRC, Chiba, Japan) under accession number NITE BP-03696. A single colony was selected from an MRS agar plate and cultured in MRS broth (Difco, BD, United States) at 32 °C for 16 h with shaking at 100 rpm. The activated culture was then scaled up in 1 L of MRS medium with 0.1% inoculation and incubated under the same conditions. Afterward, the fermented broth was centrifuged at 25 °C at 5,000 rpm (Heraeus Megafuge 40R, Thermo Fisher Scientific Inc., United States) for 10 min to collect the bacterial pellet. The pellet was washed with reverse osmosis (RO) water, mixed with 20%(w/v) skim milk powder, and freeze-dried (FD24, Kingmech Scientific, Taoyuan, Taiwan) to obtain a live bacterial powder containing approximately 4 × 10^1^1 colony-forming units (CFU) per gram, as determined by the plate counting method. For the preparation of the heat-killed GKEX sample (HK-GKEX), the washed bacterial pellet was resuspended in an equal volume of RO water, autoclaved at 121 °C for 15 min, and then freeze-dried. To prepare the freeze-killed GKEX sample (FK-GKEX), the washed bacterial pellet was resuspended in RO water, frozen at −20 °C for 3 days, and thawed at room temperature. This freeze–thaw cycle was repeated three times before processing into the powder by freeze-drying. All the bacterial powders were stored at 4 °C until use in subsequent animal experiments.

### 2.2 Animals and experimental designs

**Figure 1:**
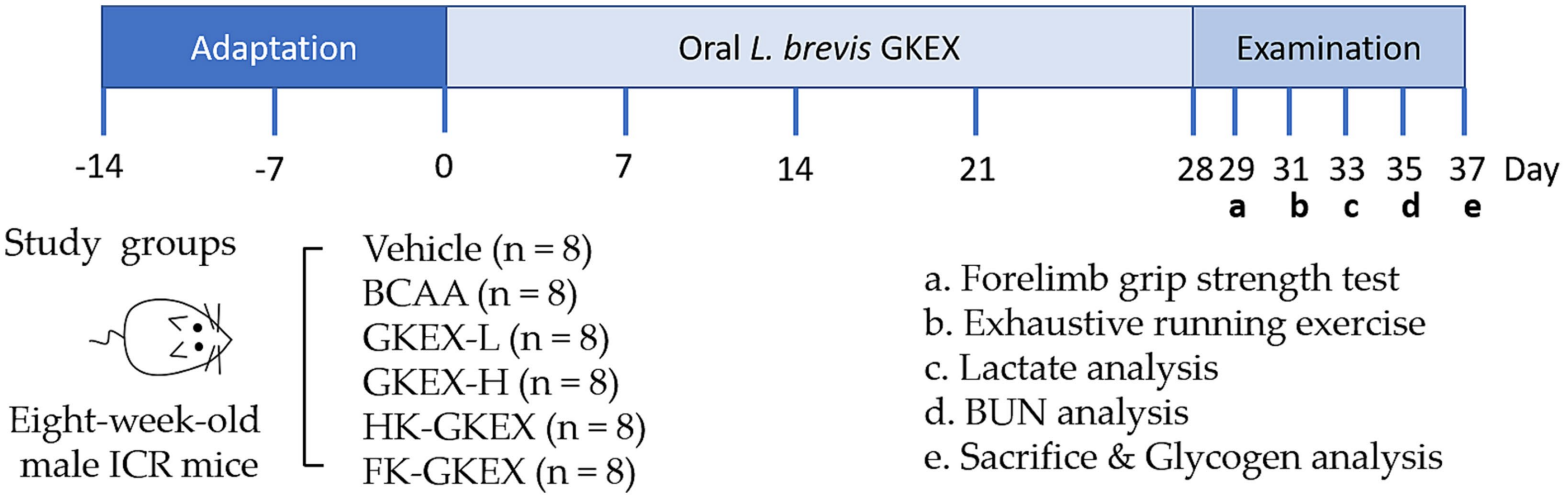
Experimental procedure.

Eight-week-old male ICR mice with an average weight of approximately 30 g were sourced from BioLASCO Taiwan (Yi-Lan Breeding Center, Yi-Lan, Taiwan). The Institutional Animal Care and Use Committee of the National Taiwan Sport University (IACUC of NTSU) approved (IACUC-11108) all animal experiments conducted. All animals were provided LabDiet^®^ 5,001 (PMI Nutrition International, Purina Mills, MO, United States) and distilled water ad libitum. The room temperature was maintained at 24 ± 2 °C with a humidity of 65 ± 5% and a 12-h light–dark cycle. A 2-week acclimation period was provided during which the mice were subjected to adaptive training to minimize stress and familiarize them with treadmill use. The adaptive training protocol consisted of placing the mice on a stationary treadmill on days 1–3, followed by light running at a speed of 5–10 m/min for 10 min/day from days 4 to 7, with gradual increases in duration and intensity over the second week to prepare them for later endurance testing. A total of 48 mice were randomly divided into 6 groups, with each group containing 8 mice: vehicle (water only) and branched-chain amino acids (BCAA, positive control, 1,500 mg/day (14)). Feeding doses of different preparations of *L. brevis* GKEX groups in mice were based on the human-to-mouse conversion factor of 12.3 (15): low-dosage *L. brevis* GKEX (GKEX-L) (0.0615 mg/day, equivalent to 10 mg/day in a human with 60 kg), high-dosage *L. brevis* GKEX (GKEX-H) (0.615 mg/day, equivalent to 100 mg/day in a human with 60 kg), HK-GKEX (0.615 mg/day, equivalent to 100 mg/day in a human with 60 kg), and FK-GKEX (0.615 mg/day, equivalent to 100 mg/day in a human with 60 kg). During the animal experiment, *L. brevis* GKEX was administered daily at 9 a.m. via oral gavage for four consecutive weeks. Throughout this period, the body weight, food intake, and water consumption were recorded. The intensity of exercise was gradually increased from low to high in consideration of animal welfare. Endurance fatigue analysis was conducted on days 29 to 37 of the experiment. This included analysis of forelimb grip strength, an exhaustive running test, post-exercise blood lactate and blood urea nitrogen (BUN) concentrations, as well as liver and muscle glycogen content. The experimental procedure is shown in Figure 1.

### 2.3 Forelimb grip strength

The forelimbs of the mice were placed on a tension rod (diameter 2 mm, length 7.5 cm) using a grip strength meter (Model-RX-5, Aikoh Engineering, Nagoya, Japan). During this process, the tail of the mouse was repeatedly pulled back to record the maximum grip force (peak power) as previously described (16).

### 2.4 Exhaustive running exercise

The mouse was placed on a mouse treadmill (MK680C, Muromachi Kikai, Chuo, Tokyo, Japan), set at a 15-degree incline with an initial speed of 12 m/min, increasing by 3 m/min every 2 min until the mouse falls into the shock zone (electric current of 2 Hz, 1.22 mA) for 5 s, showing no willingness to continue running forward, thereby indicating exhaustion. The time from the start to exhaustion was recorded as a measure of endurance performance (17).

### 2.5 Post-exercise blood biochemistry analysis

Unloaded swimming tests were conducted according to a previous study (18, 19). Mice swam for 10 min and rested for 20 min for lactate analysis, whereas mice swam for 90 min and rested for 60 min for BUN analysis. The swimming test was conducted in water at 28 ± 1 °C, and blood samples were immediately collected after swimming. The blood samples were centrifuged at 4 °C, 1,500 × *g* for 10 min to collect blood for analysis. Post-exercise fatigue markers, including lactate and BUN, were measured using an automatic blood biochemical analyzer (Hitachi 7,060, Hitachi, Tokyo).

### 2.6 Hematological profiles

At the end of the experiment, the mice were euthanized by 95% CO2 asphyxiation. Their blood was collected and then centrifuged at 4 °C, 1,500 × *g* for 15 min. The serum was aliquoted and analyzed for general clinical blood biochemistry, including glutamic oxaloacetic transaminase (GOT), glutamic pyruvic transaminase (GPT), creatine kinase (CK), BUN, creatinine (CREA), uric acid (UA), total cholesterol (TC), triglycerides (TG), glucose (GLU), and albumin (ALB), using a blood biochemical automatic analyzer (Hitachi 7,080, Hitachi, Tokyo, Japan).

### 2.7 Determination of tissue glycogen levels

This analytical method directly quantifies glycogen according to Chamberland and Rioux (20). Mice were sacrificed 30 min after the last feeding, and the liver and hind limb muscles were collected. After washing with physiological saline and drying, the tissues were homogenized with 5 times (w/v) tissue homogenization buffer using a Bullet Blender tissue homogenizer (Next Advance, Cambridge, MA, United States). The homogenates were then centrifuged at 4 °C, 12,000 × *g* for 15 min, and the supernatant was used for hepatic glycogen content analysis (21). Additionally, a calibration curve was prepared using a commercially available glycogen standard (Glycogen Sigma) to calculate changes in glycogen storage in the liver and muscle tissues among the different groups.

### 2.8 Analysis of gut microbiota composition

Fresh samples from the cecum of mice were collected after sacrifice and stored at −80 °C for DNA extraction using the Qiagen DNA Mini Kit (Qiagen, MD, United States). DNA was quantified using a NanoDrop ND-1000 spectrophotometer (Thermo Scientific, Wilmington, DE, United States) and maintained at −80 °C. Furthermore, the V3–V4 region of the 16S rRNA gene was amplified using the forward primer 341F (5’-TCGTCGGCAGCGTCAGATGTGTATAAGAGACAGCCTACGGGNGGCWGCAG-3′) and reverse primer 805R (5’-GTCTCGTGGGCTCGGAGATGTGTATAAGAGACAGGACTACHVGGGTATCTAATCC-3′). Subsequently, the PCR product was subjected to a second PCR amplification using the Nextera XT Index Kit (Illumina, United States) and quantified by real-time quantitative polymerase using a KAPA library quantification kit (KAPA Biosystems, United States). The Illumina HiSeq platform was applied to the V3 and V4 regions of 16S rRNA genes for taxonomic profiling of microbiota in cecums from kingdom to species, according to the Greengenes database^1^. Linear discriminant analysis effect size (LEfSe) analysis was performed to identify the differences between the abundance of taxonomic biomarkers of the gut microbiota in each group, and the results were visualized using a heatmap. Alpha diversity was assessed using the Shannon diversity index to evaluate the species richness and evenness within each group. The results were presented as boxplots to visualize differences in microbial diversity. Beta diversity using Bray–Curtis dissimilarity, unweighted UniFrac, and weighted UniFrac distances, which were visualized via constrained principal coordinates analysis (CPCoA).

### 2.9 Statistical analysis

Values are presented as mean ± SD, with each group consisting of eight mice. To assess group differences, a one-way analysis of variance (ANOVA) using Duncan’s *post hoc* test was conducted. The analysis was performed using SAS 9.0 (SAS Institute, Cary, NC, United States). Statistical differences in alpha diversity among groups were analyzed using the Kruskal–Wallis test, and for pairwise comparisons between groups, post hoc Dunn’s tests were performed. PERMANOVA accompanies CPCoA to determine whether the observed separation is statistically significant. A *p*-value of < 0.05 was considered statistically significant.

## 3 Results

### 3.1 Characteristics of mice following 4 weeks of *Levilactobacillus brevis* GKEX supplementation

**Figure 2:**
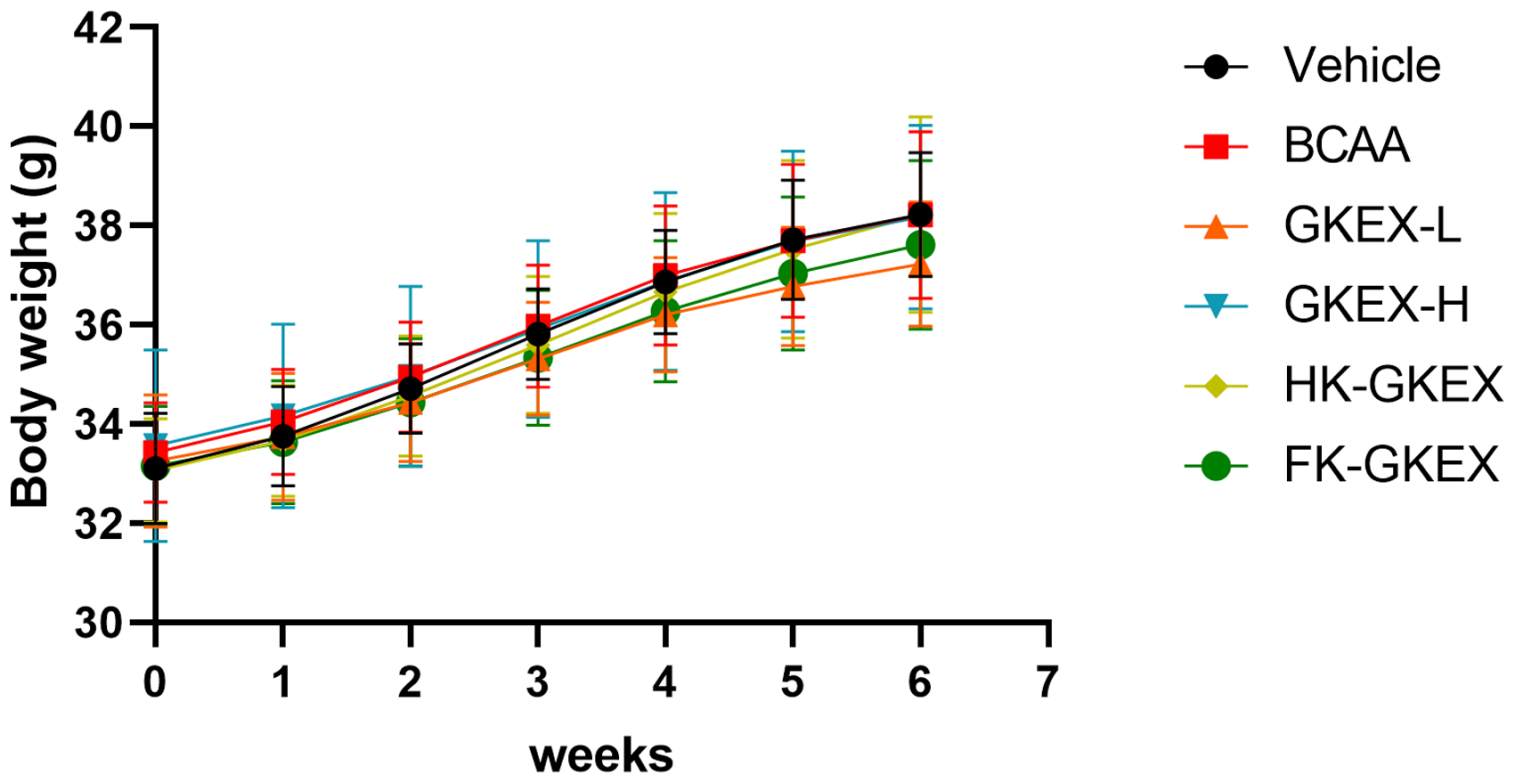
Body weights of the mice in each group during the experimental period. Data are expressed as mean ± SD (*n* = 8 mice per group). GKEX-L: low-dosage *Levilactobacillus brevis* GKEX, GKEX-H: high-dosage *L. brevis* GKEX, HK-GKEX: heat-killed *L. brevis* GKEX, and FK-GKEX: freeze-killed *L. brevis* GKEX.

During the experimental period, the body weight of mice in all six groups showed a stable increase over time, indicating no adverse effects associated with *L. brevis* GKEX supplementation. Additionally, there were no significant changes in the average body weight among the groups during the 4-week experimental period (Figure 2). As shown in Supplementary Table S1, no significant differences were observed in the average daily water and chow intake among the groups. Therefore, supplementation of different treatment methods of *L. brevis* GKEX did not significantly affect the drinking or feeding behavior of the mice. Furthermore, there were no significant differences observed in the absolute or relative weight of the tissues and organs among the groups (Supplementary Table S4), suggesting that no abnormal damage and changes were observed.

### 3.2 *Levilactobacillus brevis* GKEX supplementation enhances forelimb grip strength in ICR mice

**Figure 3:**
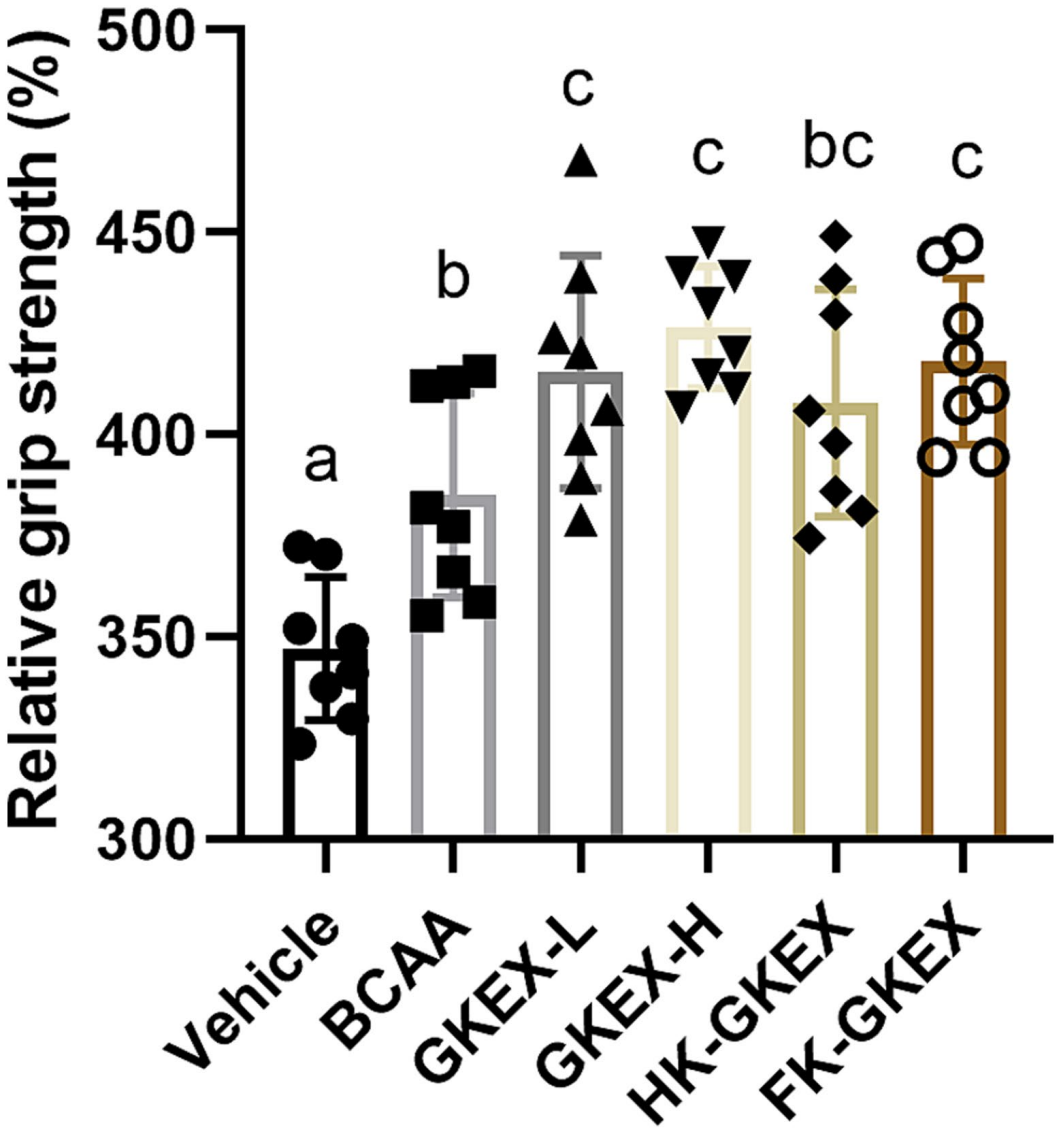
Effect of *L. brevis* GKEX supplementation on relative grip strength. Relative grip strength is calculated as forelimb grip strength divided by body weight. Data are expressed as mean ± SD (*n* = 8 mice per group). Different letters (a, b, c, d) indicate significant differences at *p* < 0.05, as determined by one-way ANOVA. GKEX-L: low-dosage *L. brevis* GKEX, GKEX-H: high-dosage *L. brevis* GKEX, HK-GKEX: heat-killed *L. brevis* GKEX, and FK-GKEX: freeze-killed *L. brevis* GKEX.

In order to determine whether *L. brevis* GKEX supplementation can enhance mouse muscle strength, the mice were fed GKEX for 4 weeks, and the forelimb grip strength of the *L. brevis* GKEX groups was compared with that of the control group. Forelimb grip strength performance may be affected by individual body weight. To adjust for this, the relative grip strength normalized to body weight (%) was calculated, as shown in Figure 3. The relative forelimb grip strength for the Vehicle, BCAA, GKEX-L, GKEX-H, HK-GKEX, and FK-GKEX groups was 347.02 ± 17.68, 385.02 ± 25.23, 415.55 ± 28.74, 426.50 ± 15.13, 407.79 ± 28.11, and 418.07 ± 20.45, respectively. Compared to the Vehicle group, the *L. brevis* GKEX group significantly increased the forelimb grip strength. Furthermore, compared to the BCAA group, the GKEX-L, GKEX-H, and FK-GKEX groups significantly increased by 1.08-fold (*p* = 0.0117), 1.11-fold (*p* = 0.0009), and 1.09-fold (*p* = 0.0069), respectively. The absolute values of grip strength are presented in Supplementary Table S2.

### 3.3 *Levilactobacillus brevis* supplementation increased exhaustive running time in ICR mice

**Figure 4:**
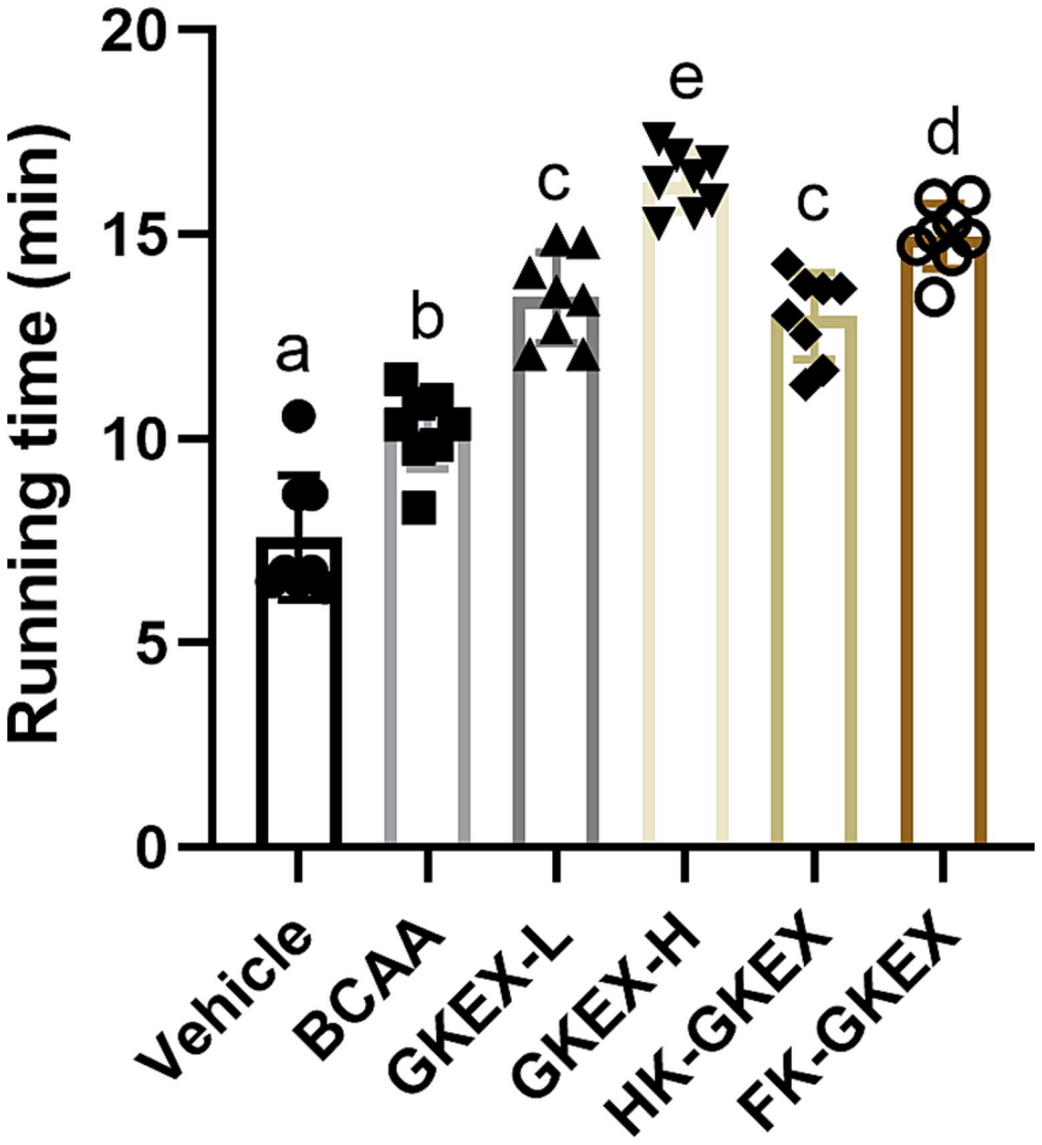
Effect of *L. brevis* GKEX supplementation on exhaustive running test. Data are expressed as mean ± SD (*n* = 8 mice per group). Different letters (a, b, c, d) indicate significant differences at *p* < 0.05, as determined by one-way ANOVA. GKEX-L: low-dosage *L. brevis* GKEX, GKEX-H: high-dosage *L. brevis* GKEX, HK-GKEX: heat-killed *L. brevis* GKEX, and FK-GKEX: freeze-killed *L. brevis* GKEX.

The running exhaustion times for the Vehicle, BCAA, GKEX-L, GKEX-H, HK-GKEX, and FK-GKEX groups were 7.59 ± 1.52, 10.23 ± 0.97, 13.45 ± 1.11, 16.28 ± 0.72, 13.00 ± 1.06, and 14.96 ± 0.80 min, respectively (Figure 4). Compared to the Vehicle group, the BCAA, GKEX-L, GKEX-H, HK-GKEX, and FK-GKEX groups showed significantly improved running exhaustion performance. Additionally, compared to the BCAA group, the GKEX-L, GKEX-H, HK-GKEX, and FK-GKEX groups showed significantly enhanced running exhaustion performance by 1.31-fold, 1.59-fold, 1.27-fold, and 1.46-fold, respectively (*p* < 0.0001). Thus, continuous supplementation with *L. brevis* GKEX effectively increased the running exhaustion performance.

### 3.4 Effect of *Levilactobacillus brevis* GKEX supplementation on serum lactate levels in post 10-min swimming test and after 20-min rest

**Figure 5:**
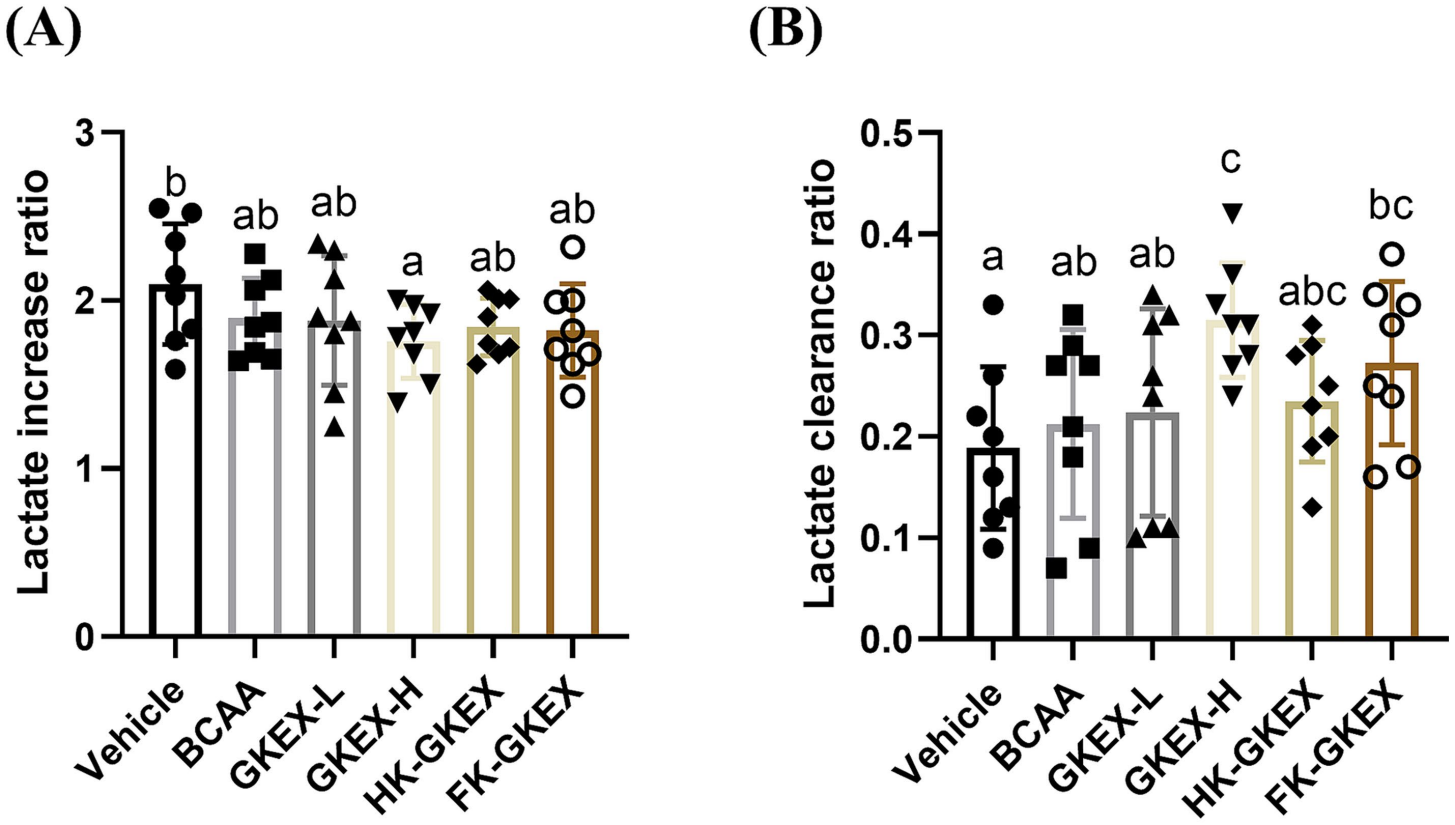
Effect of *L. brevis* GKEX supplementation on serum **(A)** lactate increase and **(B)** clearance ratio in post-10-min swimming test. The lactate increase ratio represents the ratio of the lactate level after exercise to that before exercise. The clearance ratio is calculated as the difference between the lactate level after swimming and that after 20 min of rest, divided by the lactate level after swimming. Data are expressed as mean ± SD (*n* = 8 mice per group). Different letters (a, b, c, d) indicate significant differences at *p* < 0.05, as determined by one-way ANOVA. GKEX-L: low-dosage *L. brevis* GKEX, GKEX-H: high-dosage *L. brevis* GKEX, HK-GKEX: heat-killed *L. brevis* GKEX, and FK-GKEX: freeze-killed *L. brevis* GKEX.

The serum lactate levels were measured before swimming, after 10 min of swimming, and after 20 min of rest, followed by swimming. The absolute values of lactate were recorded at different timepoints in Supplementary Table S3. Before swimming, there were no significant differences in serum lactate levels between the Vehicle, BCAA, GKEX-L, GKEX-H, HK-GKEX, and FK-GKEX groups. After 10 min of swimming, the lactate increase ratio was calculated based on the lactate levels before and 10 min after the exercise. Compared to the Vehicle group, the GKEX-H group showed a significant decrease of 1.2-fold (*p* = 0.0213) in the lactate increase rate (Figure 5A).

The lactate clearance ratio, determined following a 20-min rest period after exercise, was used to assess lactate recovery. Compared to the Vehicle group, GKEX-H and FK-GKEX groups significantly increased the lactate clearance ratio. Furthermore, compared to the BCAA group, the supplementation of the GKEX-H significantly increased the lactate clearance ratio by 1.48-fold (*p* = 0.0145) (Figure 5B).

### 3.5 Effect of *Levilactobacillus brevis* supplementation on blood urea nitrogen levels after a 90-min swimming test followed by a 60-min rest

**Figure 6:**
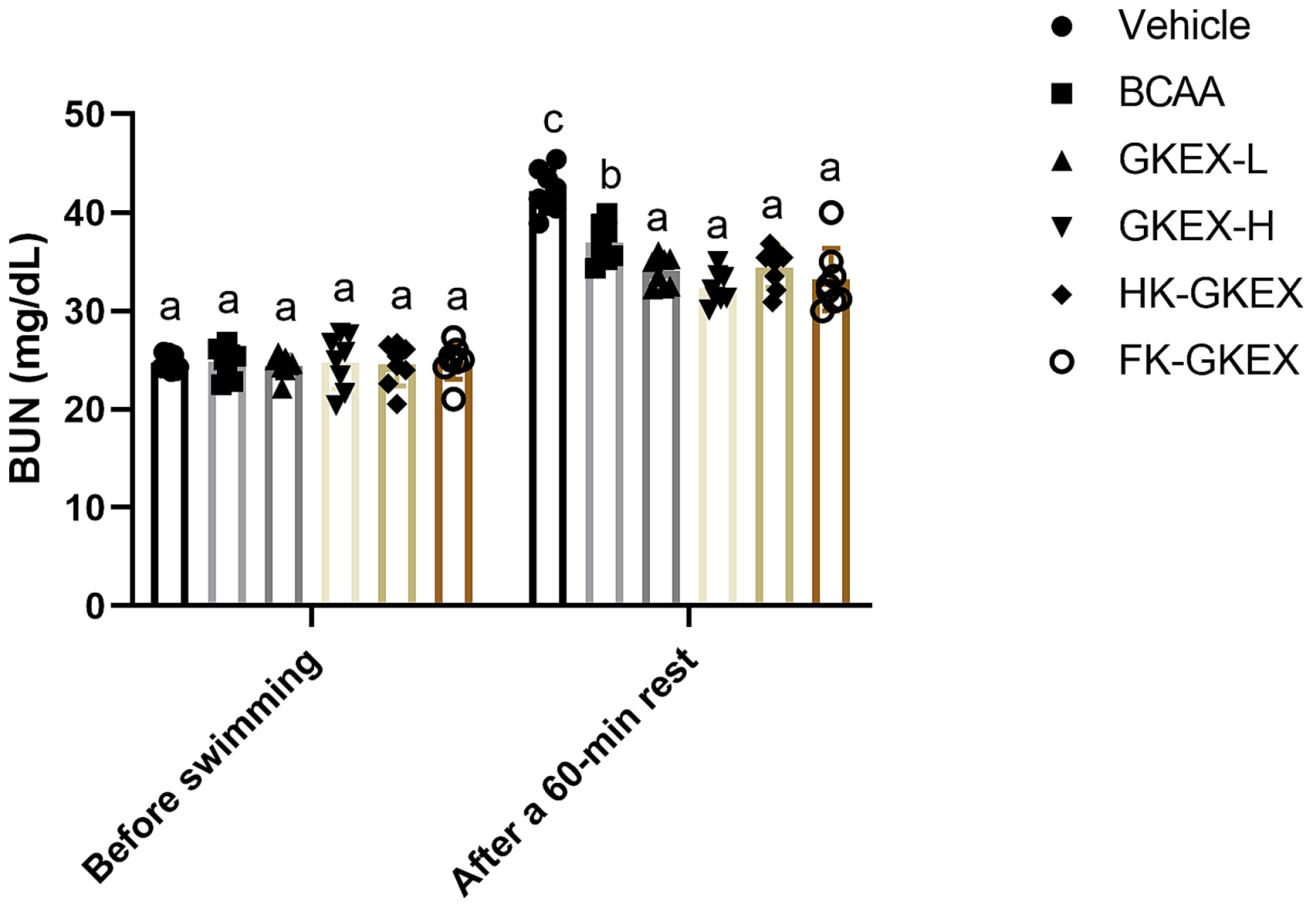
Effect of *L. brevis* GKEX supplementation on blood urea nitrogen levels after a 90-min exercise followed by a 60-min rest. Data are expressed as mean ± SD (*n* = 8 mice per group). Different letters (a, b, c, d) indicate significant differences at *p* < 0.05, as determined by one-way ANOVA. GKEX-L: low-dosage *L. brevis* GKEX, GKEX-H: high-dosage *L. brevis* GKEX, HK-GKEX: heat-killed *L. brevis* GKEX, and FK-GKEX: freeze-killed *L. brevis* GKEX.

As shown in Figure 6, BUN concentrations following a 90-min swimming test were measured in each group of mice. The BUN concentrations in the Vehicle, BCAA, GKEX-L, GKEX-H, HK-GKEX, and FK-GKEX groups showed no significant differences among the six groups.

Additionally, BUN concentrations after a 90-min swimming test followed by a 60-min rest were tested for each group of mice. Compared to the Vehicle group, supplementation with *L. brevis* GKEX significantly reduced BUN concentration. Furthermore, compared to the BCAA group, supplementation with GKEX-L, GKEX-H, HK-GKEX, and FK-GKEX significantly decreased BUN concentrations by 1.09-fold (*p* = 0.0094), 1.14-fold (*p* < 0.0001), 1.07-fold (*p* = 0.0217), and 1.11-fold (*p* = 0.0011), respectively.

### 3.6 *Levilactobacillus brevis* supplementation increases hepatic and muscular glycogen contents

**Figure 7:**
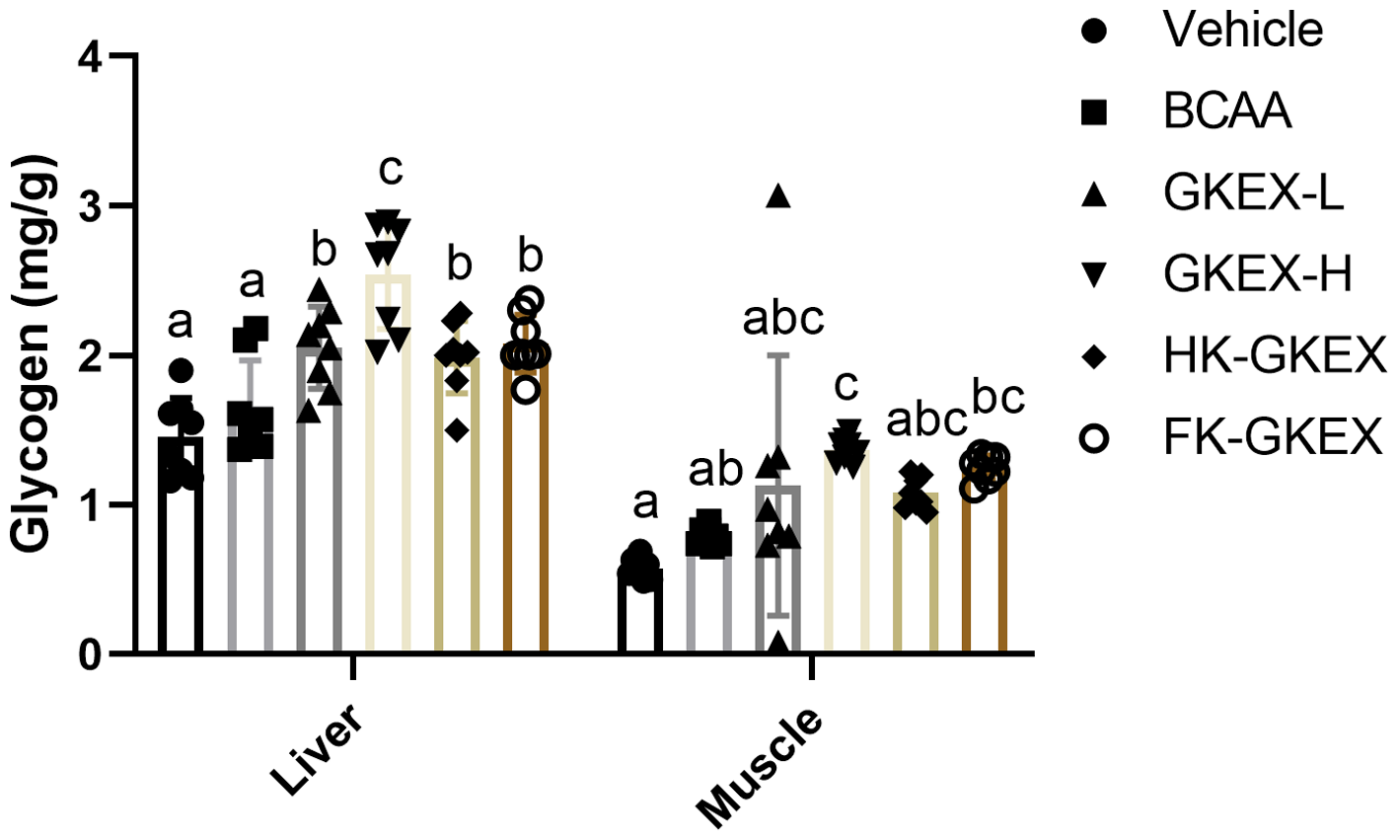
Effect of *L. brevis* GKEX supplementation on liver and muscular glycogen content. Mice were pretreated with vehicle, BCAA, GKEX-L, GKEX-H, HK-GKEX, and FK-GKEX for 28 days. Data are expressed as mean ± SD (*n* = 8 mice per group). Different letters (a, b, c, d) indicate significant differences at *p* < 0.05, as determined by one-way ANOVA. GKEX-L: low-dosage *L. brevis* GKEX, GKEX-H: high-dosage *L. brevis* GKEX, HK-GKEX: heat-killed *L. brevis* GKEX, and FK-GKEX: freeze-killed *L. brevis* GKEX.

Hepatic and muscular glycogen contents are shown in Figure 7. The hepatic glycogen content in the Vehicle, BCAA, GKEX-L, GKEX-H, HK-GKEX, and FK-GKEX groups was 1.45 ± 0.26, 1.66 ± 0.31, 2.05 ± 0.28, 2.54 ± 0.36, 1.99 ± 0.24, and 2.08 ± 0.19 (mg/g), respectively. Compared to the Vehicle group, the *L. brevis* GKEX group showed significantly increased hepatic glycogen content. In addition, compared to the BCAA group, the GKEX-L, GKEX-H, HK-GKEX, and FK-GKEX groups significantly increased hepatic glycogen content by 1.23-fold (*p* = 0.0074), 1.53-fold (*p* < 0.0001), 1.20-fold (*p* = 0.0227), and 1.25-fold (*p* = 0.0044), respectively. On the other hand, the muscular glycogen content in the Vehicle, BCAA, GKEX-L, GKEX-H, HK-GKEX, and FK-GKEX groups was 0.57 ± 0.07, 0.79 ± 0.06, 1.13 ± 0.87, 1.37 ± 0.08, 1.08 ± 0.10, and 1.25 ± 0.08 (mg/g), respectively. Compared to the Vehicle group, the GKEX-H and HK-GKEX groups showed significantly increased muscular glycogen content. Compared to the BCAA group, the GKEX-H group significantly increased muscular glycogen content by 1.38-fold (*p* = 0.0444).

### 3.7 Effect of *Levilactobacillus brevis* GKEX supplementation on gut microbiota

**Figure 8:**
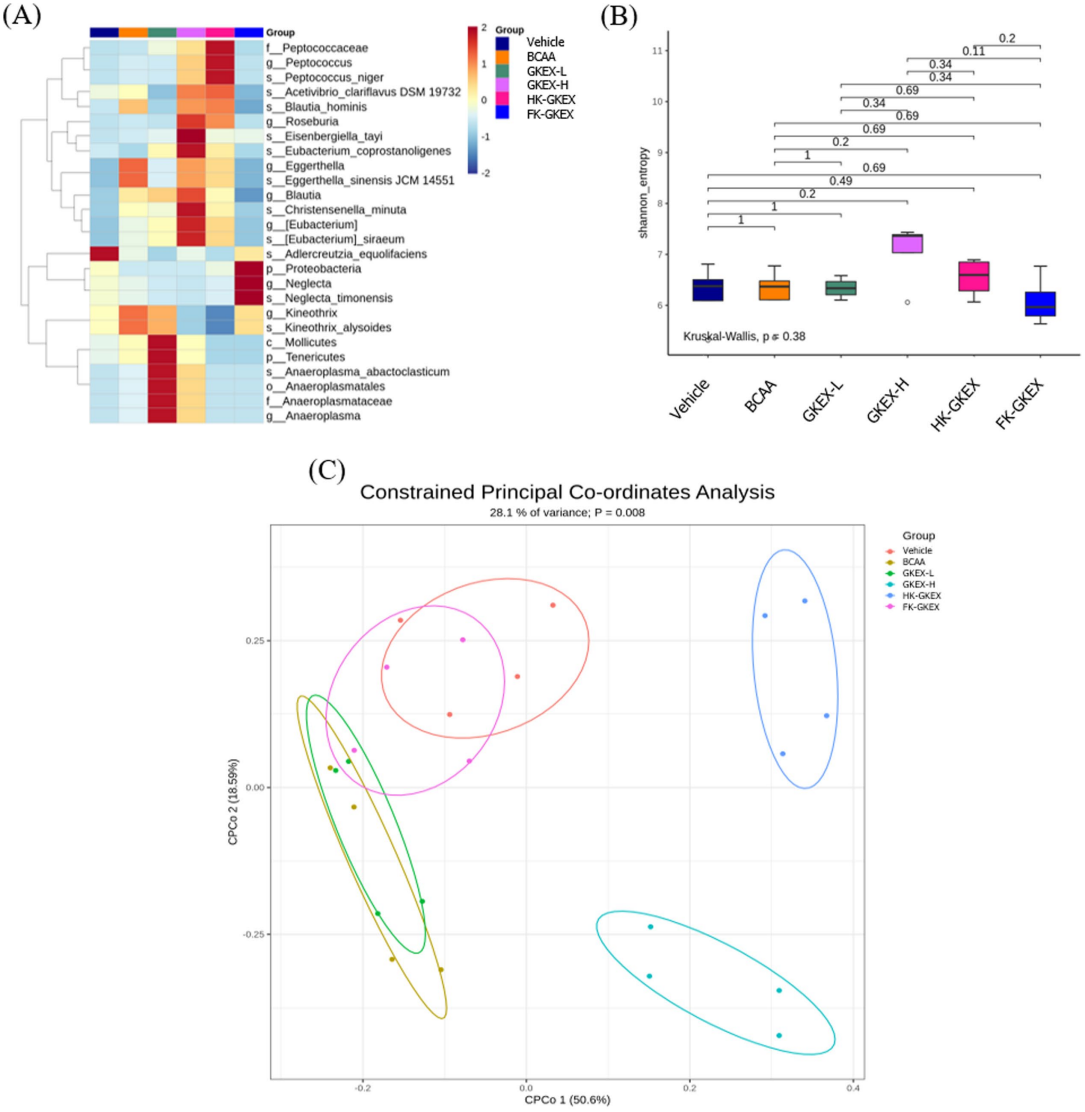
Effect of *L. brevis* supplementation on gut microbiota. **(A)** Linear discriminant analysis effect size (LEfSe) analysis of the abundance of the gut microbiota along grazing gradients. The *X*- and *Y*-axes of the thermal graph represent the experimental groups and species, respectively. p: phylum; c: class; f: family; o: order; g: genus; s: species. **(B)** The boxplot of alpha diversity using the Shannon diversity index. **(C)** Constrained principal coordinates analysis (CPCoA) based on Bray–Curtis distances of the gut microbiota profiles. Each point represents the microbial community of a sample, and the colors indicate the treatment groups. GKEX-L: low-dosage *L. brevis* GKEX, GKEX-H: high-dosage *L. brevis* GKEX, HK-GKEX: heat-killed *L. brevis* GKEX, and FK-GKEX: freeze-killed *L. brevis* GKEX.

The heatmap resulting from LEfSe analysis shows the taxonomic groups in the intestines and their abundance distribution in each group. As shown in Figure 8A, the GKEX-L group showed higher proportions of *Mollicutes* (Class), *Anaeroplasmatales* (Order), *Anaeroplasmataceae* (Family), and *Anaeroplasma* (Genus). Furthermore, an LDA score above 2 is widely considered a threshold for biologically meaningful differences in microbiota analysis, assuming that it passes the statistical significance test (Supplementary Figure S1). The abundances of *Roseburia*, *Blautia* (Family *Lachnospiraceae*), and *Eubacterium* at the genus level and that of *Christensenella minuta* (Phylum *Firmicutes*) significantly increased in the GKEX-H group compared with the vehicle group. In addition, the genus *Peptococcus* in the HK-GKEX group was significantly increased, whereas the phylum *Proteobacteria* was significantly increased in the FK-GKEX group. Supplementation with different doses of live and dead bacteria, particularly high doses of live *L. brevis* GKEX, increased the taxa of the microbiome.

Alpha diversity refers to the diversity of the microbial species within the experimental groups. The α-diversity of the gut microbiota, as measured by the Shannon index, showed no significant differences among the groups (*p* = 0.38). Pairwise comparisons also revealed no statistically significant differences (all *p* > 0.05), although a trend of increased diversity was observed in the GKEX-H and HK-GKEX groups (Figure 8B). Beta diversity was visualized by CPCoA (Figure 8C). Beta diversity measures the differences in microbial community composition between each group. BCAA and GKEX-L groups showed a high degree of overlap, indicating that their microbiota effects were similar and not markedly distinct. The FK-GKEX group overlapped partly with the Vehicle group and partly with the BCAA and GKEX-L groups, suggesting that its microbiota composition shared similarities with all three. In contrast, the GKEX-H and HK-GKEX groups were clearly separated from the others, indicating that their microbiota profiles were substantially different from both the Vehicle and other treatment groups. A clear separation between groups was observed, and PERMANOVA indicated a significant difference (*p* = 0.008) in community structure between groups.

## 4 Discussion

Probiotics can potentially promote health, exercise adaptation, and performance in athletes (22). A recent study has also indicated the potential of *Lactobacilli* in enhancing athletic performance (9). In our previous study, supplementation with *L. brevis* GKEX significantly improved forelimb grip strength and exercise endurance time in mice and maintained lower levels of blood lactate and blood urea nitrogen after exercise (11). In this study, *Levilactobacillus brevis* GKEX supplementation not only significantly improved forelimb grip strength and endurance running time but also demonstrated superior performance compared to both the vehicle and BCAA groups, particularly in lactate clearance, BUN reduction, and glycogen preservation. Moreover, unlike many studies that focus solely on live probiotics, this study highlights the beneficial effects of both live and inactivated GKEX formulations (HK-GKEX and FK-GKEX). The FK-GKEX group, in particular, revealed distinct microbiota profiles and performance outcomes, potentially due to preserved intracellular metabolites. These findings underscore the unique versatility and efficacy of GKEX across different preparation forms, which is rarely reported in previous probiotic-exercise studies. Most importantly, with the efficacy of *L. brevis* GKEX supplementation with the probiotic did not result in any abnormal changes in the weights and pathological sections of tissues and organs (Supplementary Table S4 and Supplementary Figure S2), liver function, kidney function, or blood lipid-related indices (Supplementary Table S5).

First, we evaluated the exercise performance-related tests. Forelimb grip strength was used to detect changes in motor function and muscle strength, while the exhaustive running test is a common way to measure fatigue-like behavior (17). BCAAs, as a positive control, might play a role in the central fatigue hypothesis by reducing the synthesis of brain serotonin (5-HT), which is associated with fatigue during prolonged exercise, thereby delaying the onset of fatigue symptoms (23, 24). Moreover, BCAAs may influence glycogen metabolism during prolonged exercise, potentially preserving liver and muscle glycogen levels (25). Surprisingly, the results revealed that ingestion of different types of *L. brevis* GKEX significantly enhanced both forelimb grip strength and exhaustive running time. This was evident not only when compared to the vehicle group but also when comparing the live *L. brevis* GKEX and FK-GKEX groups to the BCAA group (Figures 3, 4). This finding correlates with our prior human trials, where *L. brevis* GKEX supplementation combined with resistance exercise training resulted in increased muscle mass and grip strength (12); however, the underlying mechanism requires further investigation. According to reports, *L. plantarum* TWK10 (LP10) has also been shown to increase muscle mass and grip strength, enhance athletic performance, and further reduce physical fatigue (26). It is evident that *Lactobacillus* shows potential in enhancing athletic performance and alleviating exercise fatigue.

Lactate production plays a significant role in understanding metabolic responses to exercise and is frequently used as a fatigue marker in athletes (27, 28). During higher-intensity exercise, a significant increase occurs in lactate and hydrogen ion concentrations, leading to muscle acidification and subsequent fatigue (29). Another explanation is that the addition of lactate to muscles, both *in vivo* and *in vitro*, is associated with increased extracellular osmolarity. This causes water to exit the muscle fibers, resulting in increased intracellular ionic strength and the subsequent inhibition of force production (30). In our study, the ingestion of live *L. brevis* GKEX significantly reduced the lactate increase ratio. Furthermore, there was a significant increase in the lactate clearance ratio in the GKEX-H and FK-GKEX groups (Figure 5). Recent studies suggest that probiotic supplementation has the potential to remove and utilize blood lactate after exercise (22). The mechanism may involve lactate converting into acetyl-CoA, which is then transformed into butyryl-CoA by lactate-utilizing bacteria (31). This process is followed by the conversion of butyryl-CoA to butyrate and coenzyme A by the enzymes phosphotransbutyrylase and butyrate kinase, simultaneously generating adenosine triphosphate (ATP), thereby maintaining the host’s physiological performance during exercise (22). This suggests that live *L. brevis* GKEX has the potential to enhance metabolic capacity, delaying muscle cell inhibition and improving exercise endurance. In addition, a previous study has found that lactate from muscle enters the intestinal lumen through the bloodstream, and lactate serves as a carbon source for specific microbes, resulting in increased microbiome-derived short-chain fatty acids (SCFAs). These SCFAs, upon absorption by the host, enhance athletic performance (32). This occurrence could also explain the higher lactate clearance ratio observed after supplementation with *L. brevis* GKEX.

The elevated BUN levels indicate protein and amino acid decomposition, negatively impacting muscle contraction strength and contributing to fatigue. Thus, BUN serves as a critical biochemical marker of fatigue (33). As the exercise workload increases, BUN levels rise more significantly, leading to a slower recovery time. In our study, supplementation with different strains of *L. brevis* GKEX resulted in significantly lower BUN levels compared to the vehicle group as well as the BCAA group after prolonged exercise followed by an equivalent rest period (Figure 6). This indicates that supplementation with *L. brevis* GKEX can enhance the efficiency of physiological energy supply, thereby alleviating fatigue.

Glycogen, stored primarily in the liver and muscle tissues, serves as the main source of energy during exercise (16). Liver glycogen directly contributes to the release of glucose into the bloodstream, providing energy for the entire body, while muscle glycogen primarily serves as an energy substrate, converting it into ATP during exercise (34). We found that supplementation with different types of *L. brevis* GKEX, particularly the live GKEX group, significantly increased liver and muscular glycogen content (Figure 7). The mechanism may be related to the production of SCFAs such as propionate and butyrate by gut bacteria, which can serve as energy sources for liver and muscle cells, maintaining stable blood glucose levels to improve endurance performance during exercise (32, 35). Additionally, numerous scientific studies support the efficacy of probiotics in regulating the intestinal microbiome and influencing the levels of SCFAs in the colon (36). Specifically, lactate-utilizing bacteria can convert lactate into butyrate (37). Therefore, this process can regulate the storage and utilization of bodily energy, thereby enhancing physical endurance and exercise performance.

In this study, microbial diversity (alpha diversity) of the GKEX-H and HK-GKEX groups was elevated, and differences in species composition among groups were significant, indicating that different *L. brevis* GKEX processing methods influence the diversity of the measured community. Multiple studies have noted that an increase in fecal SCFAs produced by the gut microbiota facilitates the metabolism of carbohydrates and proteins, which further enhances skeletal muscle glucose uptake and lean muscle mass, contributing to improvements in endurance exercise capacity and muscular strength (38–40). However, we did not include the direct measurement of SCFAs in the current study, which could not elucidate the role of microbial metabolites in mediating the physiological effects of GKEX supplementation. Nevertheless, the common SCFA-producing bacteria were upregulated, including the phylum *Firmicutes*, in particular *Roseburia*, *Blautia* spp., and *Eisenbergiella tayi* of the family *Lachnospiraceae*, *Christensenella minuta* of the family *Christensenellaceae*, and *Peptococcus* of the family *Peptococcaceae* (41–45) after different doses and formulations of *L. brevis* GKEX were used. The GKEX-H and HK-GKEX groups demonstrated similar microbiota patterns, with increased levels of *Peptococcus*, *Roseburia*, *Blautia*, *Eisenbergiella tayi*, and *Eubacterium* (Figure 8). The diversity of key SCFA-producing bacteria in the live *L. brevis* GKEX-H group was higher than that in the dead *L. brevis* HK-GKEX group, which possibly resulted in better exercise performance and fatigue resistance in the GKEX-H group. Furthermore, the freeze–thaw process in the FK-GKEX group may cause the secretion of intracellular metabolites, such as enzymes, after cell membrane rupture, which does not appear in other groups. Additionally, because there is no heat treatment, some substances that are different from those in dead bacteria may be preserved. This resulted in a different microbiota pattern compared to that of the HK-GKEX and FK-GKEX groups, with an enhanced abundance of the phylum *Proteobacteria*, a potential butyrate producer (46, 47).

Overall, regardless of the type of *L. brevis* GKEX, live cells, cell debris, and cell lysates had beneficial effects on exercise performance. Compared to strains like *L. plantarum* TWK10 and *L. casei Shirota*, whose effects are primarily limited to muscle strength or immune modulation, *L. brevis* GKEX demonstrates a more comprehensive impact, from energy metabolism to microbiota modulation and systemic fatigue markers. Furthermore, SCFA levels should be measured to clarify the relationship between gut microbiota and exercise duration. Although *L. brevis* GKEX demonstrated positive effects on fatigue alleviation and exercise performance in animal models, the underlying mechanisms remain to be elucidated. In particular, genomic and metabolomic profiling of this strain has not yet been performed. Future studies should include omics-based approaches to clarify the metabolic pathways and functional genes involved in GKEX’s strain-specific ergogenic effects.

## 5 Conclusion

Our study evaluated the effects of supplementing with *L. brevis* GKEX on enhancing exercise performance and reducing fatigue. We found that after continuous supplementation with live or dead *L. brevis* GKEX for 4 weeks, mice showed improved endurance and aerobic performance during exercise, accompanied by a reduction in lactate production, even better than that in the BCAA group. Additionally, there was an enhanced lactate clearance ratio during the rest periods post-exercise and decreased BUN levels. Furthermore, there was increased glycogen content in the liver and muscles, thereby improving the physiological energy supply efficiency. Finally, supplementation promoted the growth of key SCFA-producing bacteria in the gut. In summary, we hypothesize that during exercise, the lactate produced enters the intestine via the bloodstream. The increased SCFA-producing bacteria, enhanced by *L. brevis* GKEX supplementation, can convert the lactate into SCFAs, thereby reducing exercise fatigue in the host and improving exercise performance. In this study, the modulation of fatigue-related biomarkers and energy metabolism, along with alterations in gut microbiota composition, implies an indirect influence via the gut-organ axis. Further investigations, such as targeted metabolomics and receptor pathway analysis, are needed to determine whether GKEX-derived compounds directly engage the host tissues.

## Data Availability

The original contributions presented in the study are publicly available. This data can be found here in the NCBI Sequence Read Archive (SRA) under BioProject accession number PRJNA1338346.

## Footnotes

^1^ http://greengenes.lbl.gov/

## Glossary

*L. brevis*: *Levilactobacillus brevis*
CFU: colony-forming units
HK-GKEX: Heat-killed GKEX
FK-GKEX: Freeze-killed GKEX
IACUC of NTSU: Institutional Animal Care and Use Committee of National Taiwan Sport University
BCAA: Branched-chain amino acids
GKEX-L: Low-dosage *L. brevis* GKEX
GKEX-H: High-dosage *L. brevis* GKEX
BUN: Blood urea nitrogen
GOT: Glutamic oxaloacetic transaminase
GPT: Glutamic pyruvic transaminase
CK: Creatine kinase
CREA: Creatinine
UA: Uric Acid
TC: Total Cholesterol
TG: Triglyceride
GLU: Glucose
ALB: Albumin
LEfSe: Linear discriminant analysis effect size
ANOVA: Analysis of variance
SCFAs: short-chain fatty acids
