# Supplementary material for: Effects of Levilactobacillus brevis GKEX supplementation on exercise performance and fatigue resistance in mice
Source: Front Nutr. 2025 Oct 14;12:1625645. doi: 10.3389/fnut.2025.1625645 (PMC12558773; doi:10.3389/fnut.2025.1625645)
Supplement: Supplementary file 1 [file Supplementary_file_1.docx]

Table S1. Measurements of body weight, water intake, and chow diet consumption in ICR mice over the 4-week supplementation period.

Body weight (g) was recorded weekly, while water intake (mL/day), and chow diet consumption (g/day) were recorded daily throughout the experimental period.

Data are expressed as mean ± SD (n = 8 mice per group). Different letters (a, b, c, d) indicates significant difference at p < 0.05 as determined by one-way ANOVA.

GKEX-L: low-dosage *L. brevis* GKEX, GKEX-H: high-dosage *L. brevis* GKEX, HK-GKEX: heat-killed *L. brevis* GKEX, and FK-GKEX: freeze-killed *L. brevis* GKEX, wk: week, BW: body weight.

| **Characteristics** | **Vehicle** | **BCAA** | | **GKEX-L** | | **GKEX-H** | | **HK-GKEX** | | | | **FK-GKEX** |
| --- | --- | --- | --- | --- | --- | --- | --- | --- | --- | --- | --- | --- |
| **Initial BW (g)** | 33.10±1.12 ^a^ | 33.43±1.00 ^a^ | | 33.25±1.33 ^a^ | | 33.56±1.93 ^a^ | | 33.06±1.05 ^a^ | | 33.15±1.21 ^a^ | | |
| **1st wk BW** | 33.76±1.00 ^a^ | 34.05±1.07 ^a^ | | 33.74±1.28 ^a^ | | 34.17±1.85 ^a^ | | 33.67±1.12 ^a^ | | 33.64±1.24 ^a^ | | |
| **2nd wk BW** | 34.71±0.90 ^a^ | 34.95±1.11 ^a^ | | 34.44±1.19 ^a^ | | 34.96±1.81 ^a^ | | 34.56±1.21 ^a^ | | 34.44±1.29 ^a^ | | |
| **3rd wk BW** | 35.81±0.91 ^a^ | 35.98±1.23 ^a^ | | 35.32±1.14 ^a^ | | 35.91±1.78 ^a^ | | 35.60±1.38 ^a^ | | 35.34±1.36 ^a^ | | |
| **4th wk BW** | 36.87±1.05 ^a^ | 37.00±1.40 ^a^ | | 36.20±1.15 ^a^ | | 36.88±1.79 ^a^ | | 36.66±1.59 ^a^ | | 36.28±1.42 ^a^ | | |
| **5th wk BW** | 37.71±1.20 ^a^ | 37.70±1.55 ^a^ | | 36.78±1.19 ^a^ | | 37.69±1.82 ^a^ | | 37.53±1.79 ^a^ | | 37.03±1.55 ^a^ | | |
| **Final BW (g)** | 38.23±1.25 ^a^ | 38.21±1.69 ^a^ | | 37.23±1.25 ^a^ | | 38.18±1.85 ^a^ | | 38.23±1.97 ^a^ | | 37.61±1.70 ^a^ | | |
| **Water intake** (mL/mouse/day) | 7.38±0.49 ^a^ | 7.30±0.38 ^a^ | | 7.35±1.18 ^a^ | | 7.44±0.87 ^a^ | | 7.33±0.61 ^a^ | | 7.31±0.45 ^a^ | | |
| **Chow 5001**  **Diet** intake (g/mouse/day) | 8.04±0.59 ^a^ | | 8.03±0.49 ^a^ | | 8.06±0.81 ^a^ | | 8.02±0.84 ^a^ | | 8.01±0.61 ^a^ | | 8.11±0.61 ^a^ | |

Table S2.

Absolute grip strength and relative grip strength among groups.

Relative grip strength is calculated by forelimb grip strength divided by body weight.

Data are expressed as mean ± SD (n = 8 mice per group). Different letters (a, b, c, d) indicates significant difference at p < 0.05 as determined by one-way ANOVA.

GKEX-L: low-dosage *L. brevis* GKEX, GKEX-H: high-dosage *L. brevis* GKEX, HK-GKEX: heat-killed *L. brevis* GKEX, and FK-GKEX: freeze-killed *L. brevis* GKEX, GS: grip strength.

|  | | **Vehicle** | | **BCAA** | | **GKEX-L** | | **GKEX-H** | | **HK-GKEX** | **FK-GKEX** |
| --- | --- | --- | --- | --- | --- | --- | --- | --- | --- | --- | --- |
| **Grip Strength (g)** | 130.75±4.98 ^a^ | | 144.88±5.89 ^b^ | | 152.63±8.23 ^c^ | | 160.63±7.33 ^d^ | | 152.63±4.98 ^c^ | | 154.63±5.42 ^cd^ |
| **Relative GS (%)** | | 347.02±17.68 ^a^ | | 385.02±25.23 ^b^ | | 415.55±28.74 ^c^ | | 426.50±15.13 ^c^ | | 407.79±28.11 ^bc^ | 418.07±20.45 ^c^ |

Table S3. Absolute lactate levels at different timepoint in the swimming test.

The lactate increase ratio represents the ratio of the lactate level after exercise (B) to that before (A) exercise. The clearance ratio is calculated by the difference between the lactate level after swimming (B) and that after 20 minutes of rest (C), divided by the lactate level after swimming (B).

Data are expressed as mean ± SD (n = 8 mice per group). Different letters (a, b, c, d) indicates significant difference at p < 0.05 as determined by one-way ANOVA.

GKEX-L: low-dosage *L. brevis* GKEX, GKEX-H: high-dosage *L. brevis* GKEX, HK-GKEX: heat-killed *L. brevis* GKEX, and FK-GKEX: freeze-killed *L. brevis* GKEX.

| **Characteristics** | **Vehicle** | **BCAA** | **GKEX-L** | **GKEX-H** | **HK-GKEX** | **FK-GKEX** |
| --- | --- | --- | --- | --- | --- | --- |
| **Pre (A)** | 3.84±0.48 ^a^ | 3.89±0.51 ^a^ | 3.86±0.55 ^a^ | 3.85±0.38 ^a^ | 3.84±0.17 ^a^ | 3.81±0.33 ^a^ |
| **Post-0 min (B)** | 7.93±0.60 ^c^ | 7.27±0.47 ^b^ | 7.10±0.78 ^ab^ | 6.70±0.29 ^a^ | 7.05±0.43 ^ab^ | 6.87±0.59 ^ab^ |
| **Post-20 min (C)** | 6.39±0.32 ^d^ | 5.70±0.67 ^b^ | 5.46±0.23 ^b^ | 4.58±0.35 ^a^ | 5.37±0.44 ^bc^ | 4.98±0.51 ^ac^ |
| **Ratio of lactate production and clearance** | | | | | | |
| **Increase ratio (B/A)** | 2.10±0.36 ^b^ | 1.89±0.24 ^ab^ | 1.88±0.38 ^ab^ | 1.76±0.22 ^a^ | 1.84±0.17 ^ab^ | 1.82±0.28 ^ab^ |
| **Clearance ratio [(B-C)/B]** | 0.19±0.08 ^a^ | 0.21±0.09 ^ab^ | 0.22±0.10 ^ab^ | 0.31±0.05 ^c^ | 0.24±0.06 ^abc^ | 0.27±0.08 ^bc^ |


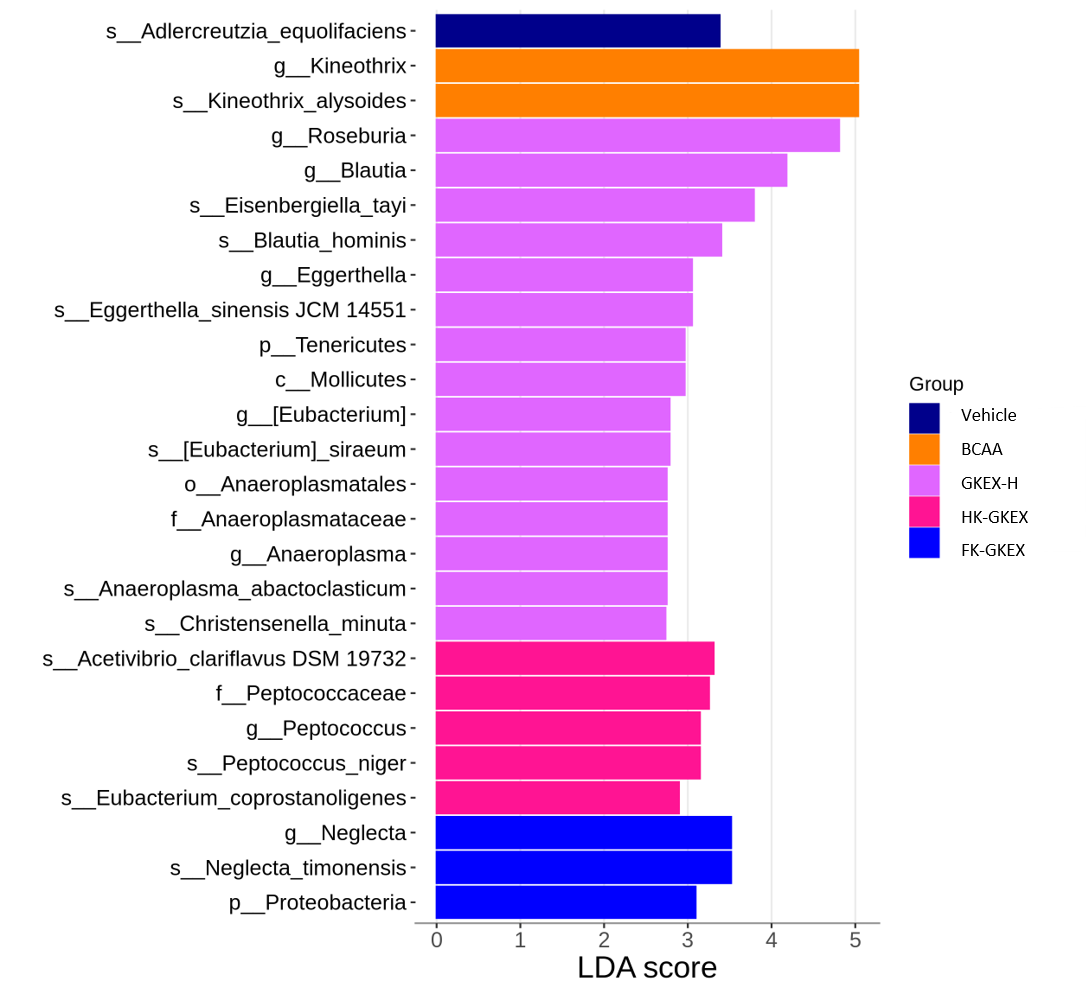


Figure S1: The barplot of all biomarkers with LDA scores exceeding 2 in each group.

GKEX-H: high-dosage *L. brevis* GKEX, HK-GKEX: heat-killed *L. brevis* GKEX, and FK-GKEX: freeze-killed *L. brevis* GKEX.

Table S4: Tissues and organs weight of the mice in each group after sacrifice. Data are expressed as mean ± SD (*n* = 8 mice per group) of weight and the percentage of tissue and organ weight in mice. Different letters (a, b, c, d) indicates significant difference at *p* < 0.05 as determined by one-way ANOVA. EFP: epididymal fat pad; GAS Muscle: ; BAT : brown adipose tissue. GKEX-L: low-dosage *L. brevis* GKEX, GKEX-H: high-dosage *L. brevis* GKEX, HK-GKEX: heat-killed *L. brevis* GKEX, and FK-GKEX: freeze-killed *L. brevis* GKEX.

| **Characteristics** | **Vehicle** | **BCAA** | **GKEX-L** | **GKEX-H** | **HK-GKEX** | **FK-GKEX** |
| --- | --- | --- | --- | --- | --- | --- |
| **Liver (g)** | 2.09±0.08 ^a^ | 2.12±0.21 ^a^ | 2.06±0.23 ^a^ | 2.09±0.12 ^a^ | 2.16±0.16 ^a^ | 2.03±0.28 ^a^ |
| **Kidney (g)** | 0.65±0.04 ^a^ | 0.64±0.03 ^a^ | 0.63±0.07 ^a^ | 0.64±0.04 ^a^ | 0.63±0.06 ^a^ | 0.65±0.09 ^a^ |
| **EFP (g)** | 0.36±0.03 ^a^ | 0.35±0.04 ^a^ | 0.37±0.04 ^a^ | 0.35±0.05 ^a^ | 0.35±0.03 ^a^ | 0.36±0.04 ^a^ |
| **GAS Muscle (g)** | 0.39±0.03 ^a^ | 0.38±0.02 ^a^ | 0.36±0.03 ^a^ | 0.37±0.02 ^a^ | 0.38±0.04 ^a^ | 0.38±0.04 ^a^ |
| **BAT (g)** | 0.09±0.01 ^a^ | 0.09±0.02 ^a^ | 0.09±0.02 ^a^ | 0.10±0.01 ^a^ | 0.10±0.02 ^a^ | 0.10±0.01 ^a^ |
| **Heart (g)** | 0.22±0.02 ^a^ | 0.23±0.04 ^a^ | 0.22±0.03 ^a^ | 0.22±0.02 ^a^ | 0.22±0.03 ^a^ | 0.21±0.02 ^a^ |
| **Lung (g)** | 0.23±0.02 ^a^ | 0.24±0.03 ^a^ | 0.23±0.04 ^a^ | 0.23±0.04 ^a^ | 0.23±0.02 ^a^ | 0.23±0.02 ^a^ |
| **Liver (%)** | 5.47±0.29 ^a^ | 5.54±0.45 ^a^ | 5.52±0.52 ^a^ | 5.47±0.38 ^a^ | 5.64±0.26 ^a^ | 5.39±0.63 ^a^ |
| **Kidney (%)** | 1.71±0.10 ^a^ | 1.68±0.10 ^a^ | 1.70±0.16 ^a^ | 1.67±0.14 ^a^ | 1.66±0.14 ^a^ | 1.71±0.18 ^a^ |
| **EFP (%)** | 0.94±0.07 ^a^ | 0.92±0.10 ^a^ | 1.00±0.10 ^a^ | 0.93±0.13 ^a^ | 0.93±0.10 ^a^ | 0.95±0.12 ^a^ |
| **GAS Muscle (%)** | 1.02±0.06 ^a^ | 1.00±0.04 ^a^ | 0.97±0.08 ^a^ | 0.98±0.06 ^a^ | 0.99±0.08 ^a^ | 1.02±0.12 ^a^ |
| **BAT (%)** | 0.22±0.04 ^a^ | 0.23±0.05 ^a^ | 0.25±0.04 ^a^ | 0.25±0.03 ^a^ | 0.25±0.05 ^a^ | 0.25±0.03 ^a^ |
| **Heart (%)** | 0.57±0.05 ^a^ | 0.60±0.11 ^a^ | 0.60±0.07 ^a^ | 0.58±0.09 ^a^ | 0.57±0.08 ^a^ | 0.55±0.07 ^a^ |
| **Lung (%)** | 0.61±0.05 ^a^ | 0.63±0.08 ^a^ | 0.62±0.09 ^a^ | 0.61±0.10 ^a^ | 0.59±0.02 ^a^ | 0.61±0.04 ^a^ |

Figure S2: The pathological sections of the mice in each group after sacrifice. (A) liver, (B) gastrocnemius muscle, and (C) epididymis adipose tissues. GKEX-L: low-dosage *L. brevis* GKEX, GKEX-H: high-dosage *L. brevis* GKEX, HK-GKEX: heat-killed *L. brevis* GKEX, and FK-GKEX: freeze-killed *L. brevis* GKEX.


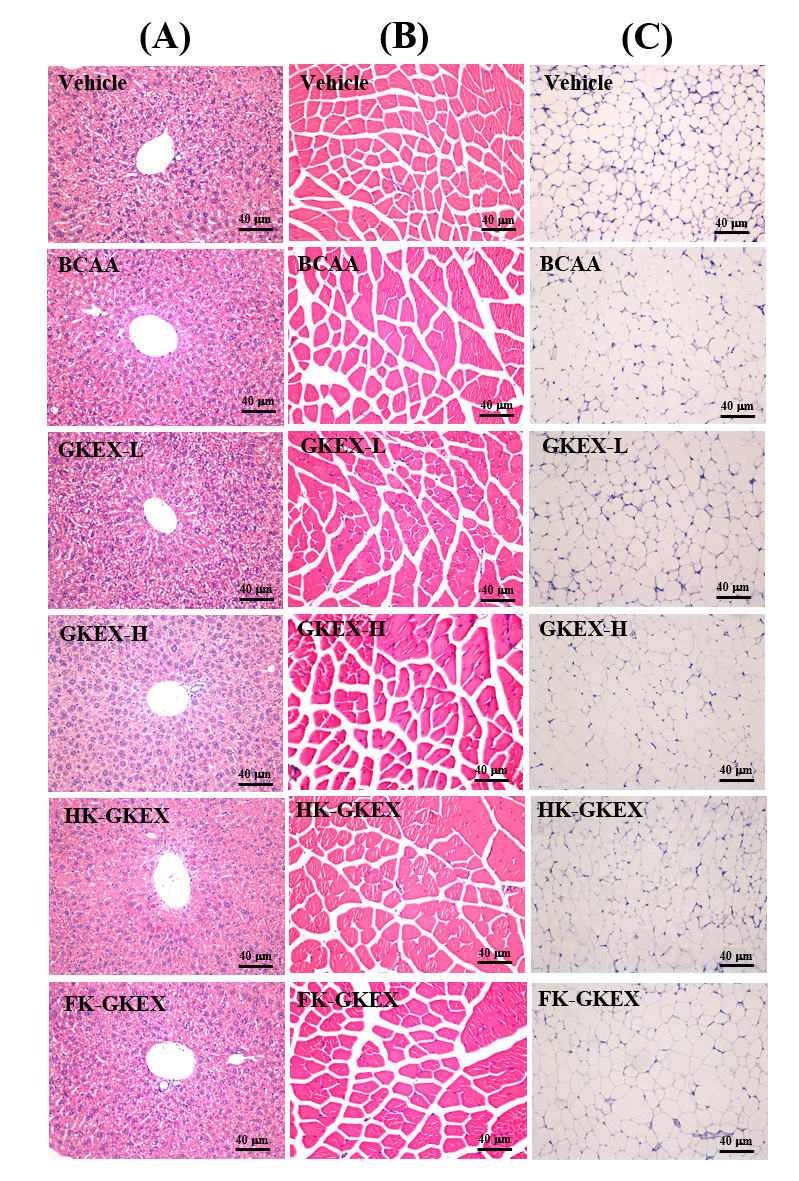


Table S5: Blood biochemical analysis after sacrifice. Data are expressed as mean ± SD (n = 8 mice per group). Different letters (a, b, c, d) indicates significant difference at *p* < 0.05 as determined by one-way ANOVA. GOT: glutamic oxaloacetic transaminase; GPT: glutamic pyruvic transaminase; CK: creatine kinase; BUN: blood urea nitrogen; CREA: creatinine; UA: uric acid; TC: total cholesterol; TG: triglyceride; GLU: glucose; ALB: albumin. GKEX-L: low-dosage *L. brevis* GKEX, GKEX-H: high-dosage *L. brevis* GKEX, HK-GKEX: heat-killed *L. brevis* GKEX, and FK-GKEX: freeze-killed *L. brevis* GKEX.

| **Characteristics** | **Vehicle** | **BCAA** | **GKEX-L** | **GKEX-H** | **HK-GKEX** | **FK-GKEX** |
| --- | --- | --- | --- | --- | --- | --- |
| **GOT(U/L)** | 67.75±11 ^a^ | 69.25±9.50 ^a^ | 68.25±8.65 ^a^ | 68.88±4.91 ^a^ | 69.25±5.18 ^a^ | 69.13±5.62 ^a^ |
| **GPT(U/L)** | 48.88±6.79 ^a^ | 47.13±8.37 ^a^ | 42.75±6.07 ^a^ | 42.13±12.16 ^a^ | 45.25±2.71 ^a^ | 47.88±7.66 ^a^ |
| **CK(U/L)** | 254.13±17.02 ^a^ | 247.88±25.38 ^a^ | 257.63±25.08 ^a^ | 257.00±26.16 ^a^ | 258.13±27.05 ^a^ | 253.38±25.15 ^a^ |
| **BUN(mg/dL)** | 23.19±2.05 ^a^ | 23.06±2.10 ^a^ | 21.89±2.76 ^a^ | 22.54±2.89 ^a^ | 23.48±1.25 ^a^ | 23.54±1.85 ^a^ |
| **CREA(mg/dL)** | 0.36±0.02 ^a^ | 0.35±0.02 ^a^ | 0.34±0.02 ^a^ | 0.35±0.02 ^a^ | 0.34±0.02 ^a^ | 0.34±0.02 ^a^ |
| **UA(mg/dL)** | 1.80±0.55 ^a^ | 1.71±0.66 ^a^ | 1.75±0.51 ^a^ | 1.65±0.26 ^a^ | 1.84±0.17 ^ab^ | 1.88±0.21 ^a^ |
| **TC(mg/dL)** | 137.25±19.57 ^a^ | 144.50±17.11 ^a^ | 135.00±24.82 ^a^ | 150.75±11.90 ^a^ | 145.00±20.02 ^a^ | 146.88±13.54 ^a^ |
| **TG(mg/dL)** | 178.50±37.21 ^a^ | 151.13±39.06 ^a^ | 155.88±17.60 ^a^ | 144.25±27.64 ^a^ | 166.63±44.98 ^a^ | 164.50±48.98 ^a^ |
| **GLU(mg/dL)** | 230.50±25.21 ^a^ | 220.75±11.62 ^a^ | 224.50±28.82 ^a^ | 223.13±39.52 ^a^ | 235.25±23.52 ^a^ | 229.75±36.41 ^a^ |
| **ALB(g/dL)** | 3.15±0.15 ^a^ | 3.07±0.16 ^a^ | 3.14±0.17 ^a^ | 3.07±0.11 ^a^ | 3.09±0.16 ^a^ | 3.10±0.10 ^a^ |
